# Differences in Inflammatory Genetic Profiles in Periodontitis Associated with Genetic and Immunological Disorders: A Systematic Review

**DOI:** 10.3390/biomedicines13122851

**Published:** 2025-11-21

**Authors:** Luis Astolfi-Labrador, Álvaro Cabezas-Corado, Daniel Torres-Lagares, María Baus-Domínguez

**Affiliations:** Department of Stomatology, Faculty of Dentistry, University of Seville, 41009 Seville, Spain; luisastolfi1@gmail.com (L.A.-L.); alvarocabezascorado@gmail.com (Á.C.-C.)

**Keywords:** periodontitis, genetic disorders, immune dysregulation, cytokines, gene expression, down syndrome, Papillon–Lefèvre syndrome, leukocyte adhesion deficiency

## Abstract

**Background**: Periodontitis is a multifactorial inflammatory disease influenced by immune and genetic factors. Certain genetic and immunological disorders, such as Down syndrome (DS), Leukocyte Adhesion Deficiency type I (LAD-I), and Papillon–Lefèvre syndrome (PLS), are associated with early-onset and severe periodontitis. Understanding their molecular and immunological mechanisms is crucial for advancing personalized therapeutic approaches. **Methods**: A systematic review was conducted following PRISMA 2020 guidelines to compare inflammatory gene expression profiles in patients with periodontitis associated with genetic or immune-mediated disorders and those without systemic conditions. Searches were performed in PubMed, Scopus, Web of Science, and Embase for studies published between 2010 and June 2025. Eligible studies reporting cytokine profiles or inflammatory gene expression were included and analyzed. **Results**: Six case–control studies met the inclusion criteria: three on DS, two on LAD-I, and one on PLS. DS patients showed increased serum levels of IL-1 beta, TNF-alpha, IL-4, IL-10, and IFN-gamma, with dysregulation of STAT1, STAT3, and SOCS3. LAD-I was characterized by overexpression of IL-17A, IL-6, IL-23, G-CSF, CXCL2, and CXCL5, indicating IL-17–driven inflammation and excessive neutrophil activation. In PLS, cathepsin C deficiency impaired activation of the antimicrobial peptide LL-37, leading to compromised host defense and accelerated tissue breakdown. **Conclusions**: Patients with periodontitis linked to genetic or immune-mediated disorders exhibit distinct inflammatory gene expression signatures that enhance disease susceptibility and progression. Identifying these immunoinflammatory pathways may guide precision periodontal therapies, although larger, standardized studies are required to validate these findings.

## 1. Introduction

Periodontitis is a chronic, multifactorial inflammatory disease characterized by dysbiotic bacterial plaque biofilms and the progressive destruction of the tooth’s supporting structures, which can ultimately lead to tooth loss if untreated [1]. Various host factors, including immune response, anatomical characteristics, and tissue structural factors, influence the pathogenesis of this disease. The majority of these factors are determined by the host’s genetic profile, which can be modified by environmental and host behavioral determinants [2].

Periodontal disease is receiving increased attention towards the role of certain systemic disorders in modifying the host response to periodontal pathogens. Individuals with specific genetic syndromes or immune deficiencies such as Down syndrome (DS), Papillon–Lefèvre syndrome (PLS), leukocyte adhesion deficiency (LAD), or other monogenic and immunoregulatory disorders frequently present with aggressive and severe forms of periodontitis at early ages [3,4,5,6]. These clinical observations suggest that the dysregulation of key inflammatory pathways may significantly contribute to disease onset and progression in susceptible populations. This genetic or immunological disorders were described in the World Workshop 2018, Albandar et al. reviewed systemic disorders that may affect the periodontal attachment apparatus and proposed case definitions, diagnostic considerations, and classification [according to the magnitude and mechanism of their effects on the periodontium) for these diseases [2].

The genetic disorders most commonly associated with severe periodontitis studied and reviewed in the literature are, on the one hand, individuals with DS, an autosomal chromosomal abnormality characterized by an extra copy of chromosome 21 [7], which have higher prevalence and severity of periodontal disease than individuals without DS [8], due to increased susceptibility to bacterial and viral infections, autoimmune diseases, and malignancies [9,10]. Few studies have explored the impairment of the immune response in DS through the analysis of serum inflammatory mediators [7], particularly in children [11], where significant changes in the levels of Interleukin-4 (IL-4), Interleukin-10 (IL-10), Interferon gamma (IFN-gamma), and tumor necrosis factor alpha (TNF-alpha) were noted. This suggests that the altered inflammatory profile in DS may plausibly influence the periodontal health of this population [12]. A further pathology of relevance is LAD, a syndrome caused by mutations on the CD18 subunit of β2 integrins, in which neutrophils are confined to blood vessels and are absent from the periodontium. This lack of neutrophil immune surveillance and the dysregulation of neutrophil-associated homeostatic pathways may lead to periodontal tissue loss [6].

It is also essential to highlight PLS, a rare autosomal recessive disease characterized by palmar and plantar hyperkeratosis and severe periodontitis affecting primary and permanent teeth, leading to early loss of primary and permanent teeth [2,13]. Cathepsin C (CTSC) deficiency leads to impaired activation of protease three and reduced levels of the cathelicidin LL-37 peptide, thereby weakening the host’s capacity to eliminate periodontal bacteria [14].

In summary, the evidence indicates that genetic and immunological disorders profoundly alter host immune responses, thereby predisposing affected individuals to early, severe, and rapidly progressing forms of periodontitis. Understanding these mechanisms is essential for improving diagnosis, risk assessment, and the development of targeted therapeutic approaches. This study will systematically analyze and contrast inflammatory gene expression profiles in individuals with periodontitis secondary to genetic or immune-mediated disorders and in patients with periodontitis in the absence of systemic conditions, to identify distinct inflammatory pathways and associations with disease progression.

## 2. Materials and Methods

The objective of this systematic review was to systematically analyze and compare the expression of inflammatory genes in patients with periodontitis associated with genetic disorders or immunological diseases versus those with periodontitis without systemic conditions, to identify potential differences in inflammatory pathways and disease progression.

### 2.1. Focused Review Questions

The following focused questions were phrased:

1. How does the expression of inflammatory genes differ between patients with periodontitis associated with genetic or immunological disorders and those with periodontitis but no systemic conditions?
2. What specific genes or inflammatory pathways show significant variation between these groups?
3. How might these genetic differences influence disease severity, progression, and treatment response in periodontitis?

### 2.2. Methodology

The *PRISMA* (Preferred Reporting Items for Systematic Review and Meta-Analyses) checklist was followed for planning and reporting this systematic review [14,15] (Supplemental Materials S1 of Supporting Information). The authors reviewed and agreed on the protocol, which was subsequently registered in the PROSPERO database (ID CRD420251014916).

Table 1 shows the PICOS (patient, intervention, comparison, outcome, and study) framework to answer the focused questions.

### 2.3. Eligibility Criteria

#### 2.3.1. Inclusion Criteria

- Study population:
  ○ Patients diagnosed with periodontitis associated with genetic immunological diseases, as classified by the American Academy of Periodontology (AAP).
  ○ Patients diagnosed with periodontitis without systemic conditions (for comparison).
  ○ Studies analyzing inflammatory gene expression in periodontal tissues or fluids (e.g., gingival crevicular fluid, saliva, biopsy samples).
  ○ Human studies (clinical trials, cohort studies, case–control studies, and cross-sectional studies).
  ○ Studies published in English or Spanish.
  ○ Publications from 2010 onwards to ensure relevance to current molecular techniques.

- Intervention(s) or exposure(s):
  ○ Studies must investigate inflammatory gene expression in patients with periodontitis associated with genetic disorders or immunological diseases, as classified by the American Academy of Periodontology (AAP).
  ○ The analysis should include molecular markers, cytokines, or other inflammatory mediators measured in gingival tissue, gingival crevicular fluid, saliva, or blood samples.
  ○ Studies comparing gene expression profiles between patients with periodontitis with and without systemic conditions will be included.
  ○ Only studies using human participants and employing validated molecular techniques (e.g., PCR, RNA sequencing, microarrays) will be considered.

- Comparator(s) or control(s):
  ○ Patients with chronic periodontitis but without any known systemic diseases or genetic disorders, who can be used as a control group to investigate how periodontitis without an underlying systemic condition differs from periodontitis with associated genetic or immunological factors.

- Study design:
  ○ Cross-sectional studies.
  ○ Case–control studies.
  ○ Cohort studies.
  ○ Clinical trials.
  ○ Randomized Controlled Trials (RCTs).

#### 2.3.2. Exclusion Criteria

- Study population:
  ○ Studies on periodontitis unrelated to genetic or immunological disorders.
  ○ Studies that do not assess gene expressions related to inflammation.
  ○ Animal or in vitro studies without human data.
  ○ Case reports, expert opinions, or narrative reviews.
  ○ Studies with insufficient data on inflammatory markers or without a clear comparison group.

- Intervention(s) or exposure(s):
  ○ Studies that do not analyze inflammatory gene expression in periodontitis.
  ○ Research focused on non-genetic or non-immunological systemic conditions (e.g., diabetes, cardiovascular diseases) without a clear link to the AAP classification.
  ○ Studies that assess only clinical periodontal parameters without molecular or genetic analysis.
  ○ Animal or In Vitro studies without direct human data.
  ○ Studies using non-validated molecular techniques or lacking a clear methodology for gene expression analysis.
  ○ Research that does not compare patients with and without systemic disorders affecting periodontitis.

- Comparator(s) or control(s): Participants with known genetic or immunological diseases, smokers (if smoking is a confounding factor in gene expression), or those with other factors influencing inflammation or periodontal health.

- Study design:
  ○ Case reports.
  ○ Expert opinions.
  ○ Narrative reviews.

### 2.4. Search Strategy

To specifically address the PICOS question, a literature search was performed on electronic databases, including the following:

- PubMed in Medline (https://pubmed.ncbi.nlm.nih.gov/, accessed on 11 March 2025).
- Scopus (https://www.scopus.com/pages/home#basic, accessed on 11 March 2025).
- Web Of Science, WOS (https://www.webofscience.com/wos/alldb/basic-search, accessed on 11 March 2025).
- Embase (https://www.embase.com/landing?status=grey, accessed on 11 March 2025).

Results from 2010 to 30 June 2025 were evaluated. For a structured literature search, MeSH terms and free text word combinations were applied related to “periodontitis”, “genetic disorders”, “immunologic disorder”, “inflammatory gene expression”, “Down’s syndrome”, “Papillon-Lefevre syndrome”, “leukocyte adhesion deficiency”, etc. Following the electronic search, hand searches were performed, including the reference lists of included papers and relevant reviews in the field.

### 2.5. Data Extraction

Study selection was conducted by two independent reviewers (L.A.L. and A.C.C.) in the following stages:

1. Screening potentially suitable titles and abstracts against the inclusion criteria to identify potentially relevant papers, resulting in a complete database by merging studies included by at least one reviewer, and

2. Screening of the full papers identified as possibly relevant from the initial screening.

The search was conducted with the above-mentioned time restrictions. All articles retrieved from the databases were exported for management, where duplicate studies will be removed.

Electronic search and additional hand search were evaluated by two reviewers (L.A.L. and A.C.C.). Studies not meeting the inclusion criteria were excluded. Relevant data from eligible studies were systematically extracted using predefined forms. This included details such as study design, participant characteristics, interventions, outcomes, measurement instruments, and effect sizes. Discrepancies were resolved by discussion and, if necessary, a third reviewer was consulted. All relevant studies meeting the inclusion criteria were included in the final review. For the initial systematic search, the level of agreement between reviewers was calculated with κ-statistics for the first- and second-stage screening results for PICOS.

As many of the papers searched were published before the current classification of periodontal diseases, an acceptable definition of periodontitis was considered as follows: attachment loss of at least 3 mm on at least two non-adjacent teeth (diagnosed by dentist/specialist) based on disease state/severity (stage/grade of periodontitis, aggressive vs. chronic periodontitis) [16]. In cases where this was not reported, the validity of the reported periodontitis diagnosis was assessed against this gold standard.

### 2.6. Quality Assessment-Risk of Bias

Risk of bias was assessed using the Cochrane ROBINS-E checklist, which is used to determine the rigor, validity, and quality of the study methods and findings. Data were evaluated independently by at least two people (A.C.C. and L.A.L.) with a process to resolve differences. Additional information was sought from study investigators if the required information was unclear or unavailable in the study publications/reports.

### 2.7. Data Synthesis

To combine the data, a systematic approach was used, following the principles outlined in the PRISMA statement and relevant extensions.

For outcomes where quantitative synthesis was not feasible (e.g., insufficient data or heterogeneous study designs), a narrative synthesis was conducted. This involved summarizing the findings from individual studies, identifying patterns in the results, and discussing the clinical significance of the findings. This approach was also used for adverse event reporting, where different types of complications may be reported across studies.

If applicable, subgroup analysis was conducted based on factors such as age, gender, or genetic disease. Sensitivity analysis was also performed to examine the robustness of the findings, testing how different assumptions (e.g., inclusion/exclusion of certain studies) affect the results.

The findings were presented clearly and transparently, following the PRISMA guidelines. The synthesis provided a summary of the overall effects, along with any limitations or uncertainties in the evidence, to guide clinical practice and future research.

The PRISMA (Preferred Reporting Items for Systematic Review and Meta-Analyses) checklist was followed for planning and reporting this systematic review (Supplementary Materials S1 of Supporting Information) [15,17]. The authors reviewed and agreed on the protocol, which was subsequently registered in the PROSPERO database (ID CRD420251014916). Table 1 shows the PICOS (patient, intervention, comparison, outcome, and studies) to answer the focused questions.

## 3. Results

### 3.1. Study Selection and Search Results

**Figure 1:**
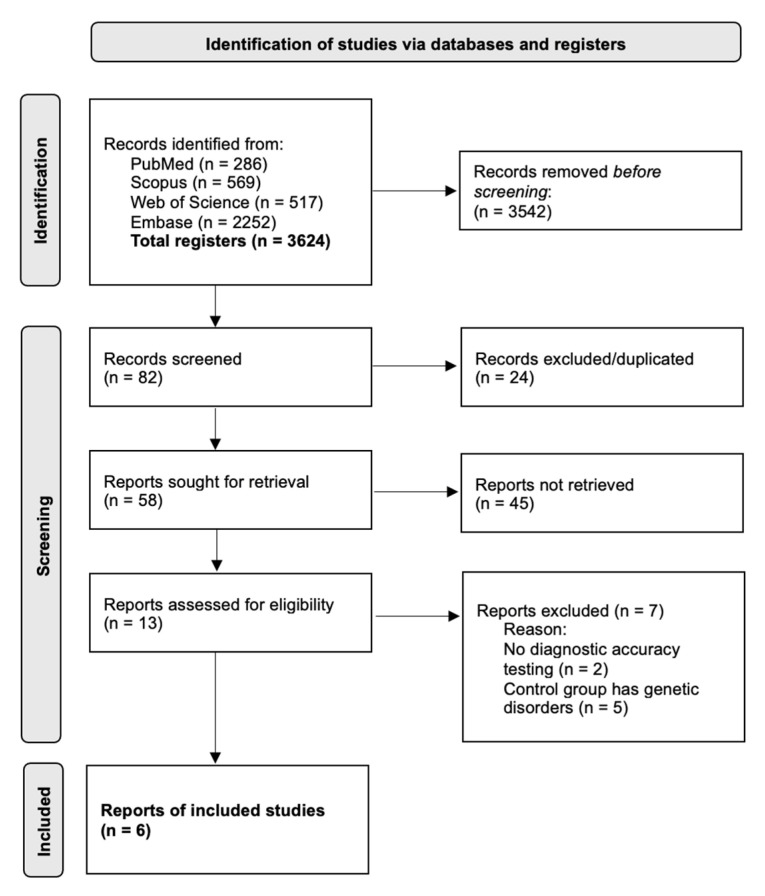
PRISMA 2020 flow diagram for new systematic reviews that included searches of databases.

An electronic search based on PICOS and an additional electronic search resulted in 286, 569, 517, and 2252 titles retrieved from PubMed, Scopus, WOS, and Embase search, respectively. Hand search resulted in seven papers. Therefore, 82 records were screened, after removal of duplicates, a total of 58 papers were included in the first screening. After a first-stage screening, 13 papers were included in the second screening. Following a second screening, six papers were deemed suitable and included. Kappa-scores (κ) were 0.988 and 0.923, respectively, at first and second screening. The reasons for excluding papers during the second stage were defined previously. Thus, six articles were included in the final selection. Figure 1 shows the flowchart of selection and inclusion methods based on the recommendations of the PRISMA (Preferred Reporting Items for Systematic Reviews and Meta-Analyses) statement published in 2020. The data extracted for each article are shown in Table 2.

### 3.2. Outcomes

A total of six observational studies met the inclusion criteria. They were classified according to the associated immune disorder (Table 2): three studies evaluated DS, two studies evaluated LAD-I, and one study evaluated PLS. All the studies presented a case–control design and analyzed biomarkers or genes related to the immunoinflammatory response in the context of periodontitis. The nomenclature of the genes and proteins mentioned in this review has been verified and standardized in accordance with the official UniProt conventions, guaranteeing terminological consistency and correct identification of each biomarker at the molecular level.

#### 3.2.1. Down Syndrome

Three studies evaluated the expression of cytokines and genes linked to the immune response in individuals with DS with and without periodontitis compared to euploid individuals with and without periodontitis [12,18,19].

Veloso et al. (2025) analyzed serum concentrations of IL-10, Interleukin-1 beta (IL-1 beta), IL-4, TNF-alpha, IFN-gamma, and Interleukin-17 (IL-17), observing elevated levels of these inflammatory mediators in subjects with Down syndrome compared to euploid controls, regardless of the severity of periodontitis [12].

Tanaka et al. (2012) examined the expression of interferon/tyrosine-protein kinase (JAK)-signal transducer and activator of transcription (STAT) axis genes (IFNG, IFNGR1, IFNGR2, IFN-alpha, IFNAR1, IFNAR2, JAK1, STAT1, IRF1). They detected a positive correlation between interferon alpha (IFNA) and probing depth (PPD ≥ 4 mm) in the group with Down syndrome and periodontitis (DS+PD). Likewise, lower expression of STAT1 (*p* < 0.02) and interferon regulatory factor 1 (IRF1) (*p* < 0.01) was identified compared to euploid controls with periodontitis [18].

On the other hand, Cavalcante Et Al. (2012) studied the expression of IL10, IL-10 receptor subunit alpha (IL-10RA), IL-10 receptor subunit beta (IL-10Rβ), JAK1, STAT3, suppressor of cytokine signaling 3 (SOCS3), intercellular adhesion molecule 1 (ICAM-1), and C-X-C motif chemokine 10 (CXCL10), demonstrating a reduction in IL10 and SOCS3 together with an increase in STAT3, which evidenced a modulation of anti-inflammatory and pro-inflammatory mediators in this group [19].

The biomarkers most frequently analyzed in studies of Down syndrome were IL-10, TNF-alpha, IL-1 beta, IL-4, IFN-gamma, and the genes STAT1, STAT3, and SOCS3, all of which are involved in the regulation of the immune response and periodontal inflammation.

#### 3.2.2. Leukocyte Adhesion Deficiency Type I

Two studies evaluated the expression of cytokines and inflammatory mediators in patients with LAD-I and periodontitis [6,20].

Moutsopoulos et al. (2015) analyzed the expression of IL-17A, IL-1 beta, IL-6, IL-23, granulocyte colony-stimulating factor (G-CSF), CXCL2, and CXCL5, observing significant IL-17A overexpression in LAD-I cases compared to healthy controls. Increases were also recorded in IL-6 and IL-23, as well as in IL-17A-dependent cytokines related to granulopoiesis and neutrophil recruitment (G-CSF, CXCL2, and CXCL5). Phenotypic analysis predominantly identified IL-17-producing CD3+ cells within the CD3+, CD8−, CD56−, TCRγδ− compartment [20].

In a previous study, Moutsopoulos et al. (2014) evaluated the cytokines IL-23 (p19 and p40), TNFα, IL-12A, IL-12B, IL1β, and IL-6, reporting that the main molecules induced by the subgingival biofilm in patients with LAD-I were the two chains of the IL-23 complex, suggesting a relevant activation of this pathway in the affected periodontal tissue [6].

The most prominent biomarkers in the LAD-I studies were IL-17A, IL-6, IL-23, G-CSF, CXCL2, and CXCL5, which were associated with amplification of the inflammatory response mediated by T lymphocytes and neutrophil recruitment.

#### 3.2.3. Papillon–Lefèvre Syndrome

A single study, conducted by Eick et al. (2014), evaluated the levels of the antimicrobial peptide LL-37 in individuals with PLS, comparing them with subjects with aggressive periodontitis, chronic periodontitis, and healthy controls. The results showed that patients with PLS presented an absence of active neutrophilic serine proteases and a lack of LL-37 maturation, findings that were associated with greater severity of periodontal disease [14].

The most relevant biomarker identified in this study was LL-37, whose deficiency was related to the alteration of antimicrobial defense mechanisms at the gingival level.

### 3.3. Quality Assessment and Risk of Bias

The assessment of the quality and risk of bias, using the Cochrane risk-of-bias tool for Non-randomized Studies of Exposure (ROBINS-E) [21], of the selected articles is summarized in Table 3. All chosen studies are classified as having a low or moderate risk of bias.

**Table 2:** Parameters of candidate diseases associated with immunologic disorders studies and periodontitis included in the systematic review.

Authors, Year of Publication	Study Type	Participants Data	Disease Associated with an Immunologic Disorder	Biomarkers and Gene Variants	Main Results
**Veloso et al. (2025)** [12]	Case-Control study	Case group (DS+, PD+, *n* = 43) Control group (E, PD+, *n* = 20)	Down Syndrome	Cytokines (IFN-gamma, IL-10, IL-17, IL-1 beta, IL-4, and TNF-alpha)	Individuals with DS exhibit elevated serum levels of inflammatory mediators, including IL-10, IL-1 beta, IL-4, and TNF-alpha, regardless of the severity of periodontitis. Furthermore, periodontitis did not correlate with changes in serum inflammatory mediator levels in the adjusted analysis, indicating that the relationship may be more nuanced.
**Moutsopoulos et al. (2015)** [20]	Case-Control study	Case group (LAD-I, *n* = 5) Control group (Healthy, *n* = 5)	Leukocyte Adhesion Deficiency Type I	Cytokines (IL-17A, IL-1β, IL-6, IL-23, G-CSF, CXCL2 and CXCL5) IL-17–producing cells	LAD-I periodontitis detected a marked up-regulation of IL17A expression compared to HP periodontitis. Cytokines linked to the induction and amplification of IL-17A expression, such as IL-1 beta, IL-6, and IL-23, were readily expressed in LAD-I periodontitis, although only IL-6 and IL-23 were significantly elevated relative to HP periodontitis. IL-17A–dependent cytokines or chemokines linked to neutrophil granulopoiesis or recruitment, such as G-CSF, CXCL2, and CXCL5, also displayed significant up-regulation in LAD-I periodontitis. Characterization of the IL-17–producing cells revealed predominantly CD3+ IL-17 producers, specifically within the CD3+, CD8−, CD56−, TCRγδ− compartment.
**Moutsopoulos et al. (2014)** [6]	Case-Control study	Case group (sLAD-I, *n* = 1; mLAD-I, *n* = 4) Control group (Healthy, *n* = 12)	Leukocyte Adhesion Deficiency Type I	Cytokines (IL-23 molecule: chain p40 and p19, TNFα, IL-12A, IL-12B, IL-23-A, IL1β and IL6)	The dominant cytokines induced in response to subgingival plaque (of health or LAD) were the two chains of the IL-23 molecule.
**Tanaka et al.** **(2012)** [18]	Case-Control study	Group 1 (DS, PD-; *n* = 19) Group 2 (E, PD+; *n* = 20) Group 3 (E, PD-; *n* = 12)	Down Syndrome	IFNG (IFNGR1, IFNGR2, IFN-alpha, IFNAR1, IFNAR2), JAK1, STAT1 and IRF1	The group with DS+PD demonstrated a strong positive correlation between IFNA mRNA levels and PPD ≥ 4 mm (0.74). Also demonstrated significantly lower expression of STAT1 (*p <* 0.02) and IRF1 (*p* < 0.01) genes in comparison with euploids during the same inflammatory stimulus of PD. For E+PD, a strong negative correlation (−0.74) was observed between CAL (mean) and IFNGR2 mRNA levels. Regarding EH, a strong negative correlation (−0.84) was observed between IFNAR2 and STAT1 mRNA levels and both PPD (mean) and CAL (mean) indices.
**Cavalcante et al. (2012)** [19]	Case-Control study	Group 1 (DS, PD-; *n* = 19) Group 2 (E, PD+; *n* = 20) Group 3 (E, PD-; *n* = 12)	Down Syndrome	IL-10 (IL-10RA, IL-10R β), JAK-1, STAT-3, SOCS-3, ICAM-1, CXCL10	It is demonstrated that the reduced expression of IL-10 is coupled with an increase of STAT-3 and a reduction of SOCS3 mRNA, indicating an essential modulation of the immune response, with attenuation of anti-inflammatory and an increase of proinflammatory mediators. This modulation may be related to the increased prevalence and severity of periodontitis in individuals with DS.
**Eick et al.** **(2014)** [14]	Case-Control study	Group 1 (PLS, *n* = 11) Group 2 (AP, *n* = 7) Group 3 (CP, *n* = 12) Group 4 (HP, *n* = 9)	Papillon–Lefèvresyndrome	LL-37	Results indicate that the lack of active neutrophil serine proteases and mature LL-37 is associated with the severity of periodontal disease in PLS patients.

***n***: sample size; **AP**: aggressive periodontitis; **CAL**: clinic attachment loss; **CP**: chronic periodontitis; **DS**: Down Syndrome; **E:** euploid; **HP**: healthy patients; **LAD-I**: Leukocyte Adhesion Deficiency Type I; **PLS**: Papillon–Lefèvre Syndrome; **PD**: periodontal disease; **PPD:** probing pocket depth; **IFN-gamma**: Interferon gamma; **IFNG**: Interferon gamma gene; **IFNGR1**: Interferon gamma receptor 1; **IFNGR2**: Interferon gamma receptor 2; **IL-10**: Interleukin 10; **IL-17/IL-17A**: Interleukin-17/Interleukin-17A; **IL-1 beta**: Interleukin 1 beta; **IL-4**: Interleukin 4; **TNF-alpha**: Tumor necrosis factor alpha; **IL-6**: Interleukin 6; **IL-23**: Interleukin 23; **IL-23-A**: Interleukin 23 subunit alpha; **IL-12B:** Interleukin 12 subunit beta; **IL-12A:** Interleukin-12 subunit alpha; **G-CSF**: granulocyte colony stimulating factor; **CXCL2**: c-x-c motif chemokine ligand 2; **CXCL5**: C-X-C motif chemokine ligand 5; **IFN-alpha**: Interferon alpha; **IFNAR1**: Interferon alpha/beta receptor 1; **IFNAR2**: Interferon alpha/beta receptor 2; **JAK1**: Tyrosine-protein kinase JAK1; **STAT1**: signal transducer and activator of transcription 1; **STAT3**: Signal transducer and activator of transcription 3; **IRF1**: Interferon regulatory factor 1; **IL-10RA**:Interleukin-10 receptor alpha; **IL-10Rβ**: Interleukin-10 receptor beta; **SOCS-3**: suppressor of cytokine signaling 3; **ICAM-1**: Intercellular adhesion molecule 1; **CXCL10**: C-X-C motif chemokine 10; **LL-37**: Cathelicidin antimicrobial peptide LL-37.

**Table 3:** Quality and bias risk evaluation using the Cochrane risk-of-bias tool for Non-randomized Studies of Exposure (ROBINS-E) [21]. The risk of bias in the included studies [6,12,14,18,19,20] was observed as low risk (green), some concerns (yellow), and high risk (red).

Risk of Bias In Non-Randomized Studies of Exposure (ROBINS-E)							
	D1	D2	D3	D4	D5	D6	D7
	Confounding	Measurement of the Exposure	Selection of Participants	Post-Exposure Interventions	Missing Data	Measurement of the Outcome	Selection of the Reported Result
Cavalcante et al. (2012) [19]							
Tanaka et al. (2012) [18]							
Eick et al. (2014) [14]							
Moutsopoulos et al. (2014) [6]							
Moutsopoulos et al. (2015) [20]							
Veloso et al. (2025) [12]							

None of the included studies achieved a low risk of bias rating, as all presented at least moderate to serious methodological limitations. The main source of bias across the evidence was identified in Domain 1 (Confounding). This domain evaluates whether differences between exposed and comparison groups—unrelated to the exposure itself—could have influenced the observed outcomes. In the context of the present review, confounding was considered particularly relevant because the included studies compared individuals with underlying genetic or immunological disorders (such as DS, PLS, or LAD-I) to systemically healthy controls. The confounders pre-specified as critical were age, sex, periodontal disease severity (probing depth, attachment loss, and bleeding on probing), oral hygiene level or bacterial plaque, use of antibiotics or anti-inflammatory medication, systemic comorbidities, and access to professional dental care. These factors are biologically and clinically associated with both exposure (the presence of the genetic/immunological condition) and outcomes (inflammatory gene expression, cytokine levels, and microbial profiles) and therefore have the potential to distort the true association.

Across studies, most authors described comparable groups regarding age and sex, and frequently excluded participants with systemic diseases or recent pharmacological treatments; however, none of the studies implemented statistical adjustment for confounders or matched participants beyond basic demographic variables. Moreover, relevant periodontal and behavioral factors such as plaque accumulation, oral hygiene practices, socioeconomic status, or long-term antibiotic prophylaxis were often not measured or reported. Consequently, the comparability between exposed and non-exposed groups remains uncertain, and the potential for residual or unmeasured confounding is high.

Regarding Domain 2 (Classification of the exposure), most studies were judged at low risk, as genetic or immunological conditions such as Down syndrome, Papillon–Lefèvre syndrome, and Leukocyte Adhesion Deficiency I were objectively confirmed through genetic or clinical criteria. However, the study by Veloso et al. (2025) [12] presented some concerns, as the classification of periodontitis subgroups relied solely on clinical parameters without standardized calibration, allowing possible misclassification.

For Domain 3 (Selection of participants), the main limitation across studies was recruitment from specialized or referral centers, resulting in small, clinically heterogeneous samples. The studies by Eick et al. (2014) [14], Moutsopoulos et al. (2014) [6], and Moutsopoulos et al. (2015) [20] were rated as having some concerns because their selective recruitment reduced representativeness and limited external validity, although inclusion criteria were consistently applied across comparison groups.

In Domain 4 (Post-exposure interventions), the genetic or immunological conditions under investigation are stable and non-modifiable; thus, post-exposure deviations were generally absent. Nonetheless, Moutsopoulos et al. (2014) [6] was judged as some concerns due to chronic or prophylactic antibiotic therapy routinely administered to LAD-I patients, which could influence both microbial composition and inflammatory outcomes.

All studies were judged to be at low risk of bias across Domains 5 to 7. No significant missing data were reported, as all samples and gene expression measurements were successfully obtained and analyzed. Outcome assessment was performed using objective, standardized molecular and microbiological techniques (e.g., RT-qPCR, ELISA, or microarray), applied equally across groups to minimize detection bias. Moreover, all studies reported outcomes as initially described in their methodologies, with no evidence of selective or incomplete reporting.

## 4. Discussion

This systematic review yields six papers that summarize the interrelationships among genetic and immunological alterations and susceptibility to periodontal disease. This study aimed to examine and compare the patterns of inflammatory gene expression in individuals with periodontitis associated with genetic or immune-mediated disorders and in those with periodontitis without systemic conditions, to identify inflammatory mechanisms and their relationship to disease progression. Across the different immunological disorders analyzed—DS, LAD-I, and PLS—significant dysregulation was observed in the expression of pro-inflammatory and regulatory cytokines, as well as in genes involved in both innate and adaptive immune responses.

According to the American Academy of Periodontology, there are numerous diseases associated with immune disorders that affect the periodontal attachment apparatus (DS, Leukocyte Adhesion Deficiency syndromes (LADs), PLS, Haim–Munk syndrome, Chediak-Higashi syndrome, Severe neutropenia, Primary immunodeficiency diseases, and Cohen syndrome) [2]. In this review, only three genetic and immunological alterations of the disease (DS, LAD-I, PLS) were studied, due to inclusion and exclusion criteria. Therefore, the analysis of this review may be more limited.

**Figure 2:**
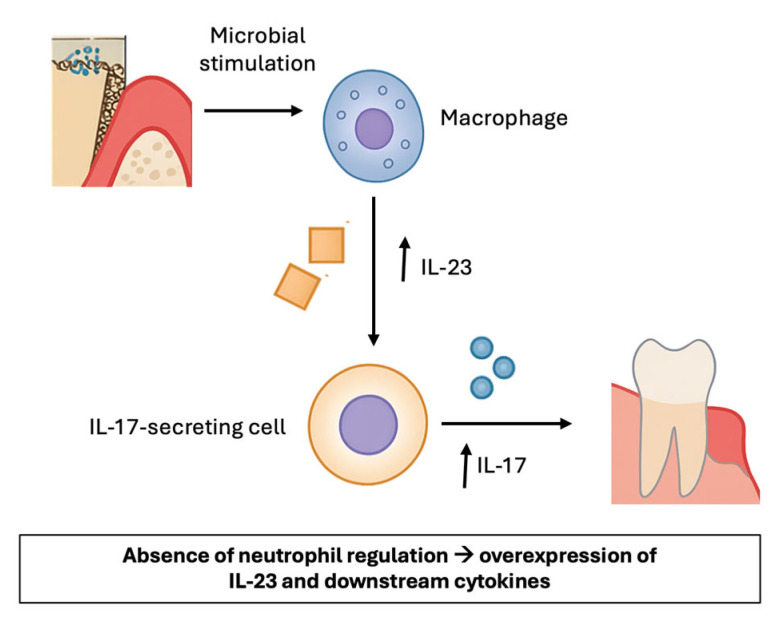
Overview of the role of IL-23 and IL-17 in LAD-I. Macrophages produce IL-23. IL-23 induces the production of IL-17 by various cells and contributes to inflammation by upregulating the production of inflammatory mediators, which induce inflammation in periodontal tissues.

Despite the scant information in the literature on LAD-I, studies emphasize the critical role of the IL-17 pathway, as shown in Figure 2 [20]. In this disorder, periodontitis has been associated with a marked upregulation of IL-17A and other related cytokines, as well as chemokines responsible for neutrophil recruitment (CXCL2, CXCL5). These findings are consistent with the proposed model, in which impaired leukocyte migration triggers a compensatory IL-17–dependent inflammatory response that, in turn, amplifies periodontal tissue destruction. Within this context, IL-23 and its subunits p40 and p19 were also shown to play a relevant role, reinforcing the notion that the IL-23/IL-17 axis is central to the immunopathogenesis of LAD-I–associated periodontitis [6,20]. Therefore, the IL-17 pathway could represent a potential target for future therapeutic research, rather than an established treatment strategy. This hypothesis is supported by the observation that patients with chronic granulomatous disease (CGD), who have defective neutrophil-driven oxygen-dependent bactericidal activity and suffer from recurrent or persistent infections, are not susceptible to periodontitis, in contrast to LAD patients [22]. It was observed that, in LAD-I patients, the identified CD3+CD8−CD56−TCRgd−IL-17 producers were presumed to be CD4+ [T helper 17 (TH17)]; however, the authors cannot rule out that these cells might in fact be a population of CD3+CD4−CD8− cells similar to the ones identified in patients with systemic lupus erythematosus or Sjögren’s syndrome as dominant sources of pathogenic IL-17 [23,24]. Altogether, these findings highlight the IL-23/IL-17 (or IL-23) axis as an intriguing immunopathogenic mechanism that warrants further investigation as a possible therapeutic target in LAD-I and other related disorders, like generalized aggressive periodontitis associated [25].

In PLS, the absence of active neutrophilic serine proteases and the antimicrobial peptide LL-37 has been identified and associated with greater severity of periodontal disease [26,27]. In this genetic disorder, we found loss-of-function mutations in the cathepsin C gene, which is highly expressed in immune cells and epithelial tissues. The cathepsin C, is a lysosomal cysteine protease that plays a key role in the activation of serine proteases in cytotoxic T cells, natural killer cells, mast cells, and neutrophils, crucial significance in immune and inflammatory defenses, the deficiency leads to impaired activation of protease three and reduced levels of the cathelicidin LL-37 peptide, thereby weakening the host’s capacity to eliminate periodontal bacteria, resulting in an oral environment highly permissive to bacterial colonization and rapid progression of periodontitis [2,14,26,28]. The subgingival microbiota in subjects with PLS comprises diverse bacterial species, including periodontal pathogens commonly associated with chronic and aggressive periodontitis, but also opportunistic pathogens [26,27]. It has also been proposed that the continuous recruitment and accumulation of hyperactive or reactive neutrophils in PLS lead to the release of elevated levels of pro-inflammatory cytokines. This process, combined with the reduced antimicrobial capacity of neutrophils, may result in a locally destructive chronic inflammatory cycle, ultimately causing severe loss of periodontal tissues [29]. Unlike DS and LAD-I, where cytokine signaling dysregulation predominates, in PLS, the underlying defect lies primarily in the innate antimicrobial response, which accounts for the early onset and severe presentation of periodontitis in affected individuals. These patients need rigorous, multidisciplinary treatment, early diagnosis of PLS, and suppression of *A. actinomycetemcomitans* to undetectable levels appear to be critical determinants of therapeutic success. Achieving these outcomes requires substantial clinical effort and patient compliance. Studies underscore the significance of timely intervention, meticulous oral hygiene maintenance, regular professional dental care, and, when indicated, the administration of systemic antibiotics [30].

Concerning DS, several studies reported an upregulation of pro-inflammatory cytokines (IL-1 beta, TNF-alpha) and a direct correlation between IFN-alpha levels and periodontal attachment loss, suggesting an exacerbated immune-inflammatory environment that may underlie the higher prevalence and severity of periodontitis in this population [12,19,31]. However, some findings contradict the hypothesis of a simple hyperinflammatory response, as reduced expression of STAT1 and IRF1, key molecules in interferon signaling, was also reported [18]. In the literature, other biomarkers have been described that might play a role in periodontal inflammation, such as soluble urokinase plasminogen activator receptor (suPAR), galectin, matrix metalloproteinase-8 (MMP-8), MMP-9, and NOD-like receptor family pyrin domain-containing protein-3 (NLRP3) inflammasome complex [32,33]. As for the NLRP3 inflammasome, it is of special interest because some preliminary evidence suggests that NLRP3 may serve as an independent predictor of disease risk during the subclinical stages of inflammatory conditions such as periodontitis and type II diabetes mellitus. Furthermore, NLRP3 has been shown to stimulate T and B lymphocytes, promoting the production of cytokines and antibodies, as well as the release of prostaglandin E2 and metalloproteases from monocytes and fibroblasts, which ultimately contribute to periodontal tissue destruction. In addition, NLRP3-mediated induction of IL-1β has been associated with osteoclast formation and neutrophil chemotaxis, processes that enhance alveolar bone resorption and impair endothelial function through a C-reactive protein (CRP)-dependent pathway, ultimately leading to an increased risk of endothelial dysfunction [34,35]. This indicates that rather than a uniform upregulation, the immune profile in DS is characterized by a complex imbalance in which elevated inflammatory mediators coexist with defects in specific regulatory pathways.

**Figure 3:**
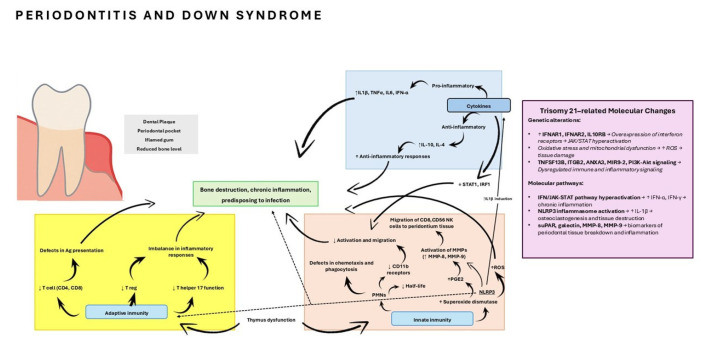
Immune function in Down syndrome. Abnormalities of the cytokines, innate, and adaptive immune system in Down syndrome predispose to periodontitis. Ag, antigen; MMP, matrix metallo proteinase; NK cell, natural killer cell; PGE2, prostaglandin E2; PMN, polymorphonuclear; Treg, T regulatory; JAK1, Tyrosine-protein kinase JAK1; STAT1, signal transducer and activator of transcription 1; TNFSF13B, tumor necrosis factor ligand superfamily member 13B gene; ITGB2, Integrin beta-2 gene; ANXA3, annexin A3; ANXA5, annexin A5; PIWIL1, Piwi-like protein 1 gene; MIR9-2, microRNA 9-2 gene; PI3K-Akt, phosphatidilinositol 3-kinase-protein kinase B; NLRP3, NOD-like receptor family pyrin domain-containing protein-3 inflammasome complex; suPAR, soluble urokinase plasminogen activator receptor; ROS, Reactive Oxygen Species; IL-1β, Interleukin 1 beta; IL-4, Interleukin 4; IL-10, Interleukin 10; IL-6, Interleukin 6; TNF-α: tumor necrosis factor alpha. Modified from Ghaffarpour et al. (2024) [36].

In the field of genetics, studies across databases compare genetic pathways only between individuals with DS with and without periodontitis. Various genes that contribute to the development of the disease have been studied, some of them with greater or lesser significance, such as tumor necrosis factor ligand superfamily member 13B gene (TNFSF13B), Integrin beta-2 gene (ITGB2), annexin A3 (ANXA3), ANXA5, Piwi-like protein 1 gene (PIWIL1), microRNA 9-2 gene (MIR9-2), or phosphatidilinositol 3-kinase-protein kinase B (PI3K-Akt) signaling pathway [31,32]. All these metabolic pathways are involved in the pathogenesis of periodontitis in DS, providing a basis for future studies on genetic susceptibility. A schematic overview of the main dysregulated molecular pathways is presented in Figure 3, modified from Ghaffarpour et al. (2024) [36]

The genetic influence on the immune-inflammatory response opens up the possibility of identifying patients susceptible to certain diseases, such as periodontitis. Recent advances in human genomics and molecular medicine are transforming our ability to understand and manage health and disease. Approximately one-third of the population variation in periodontitis is attributed to genetic factors, which is especially relevant in hereditary and immunological diseases such as DS, LAD I, and PLS, in which genetic alterations condition an immune dysfunction that increases susceptibility to periodontal destruction [37]. Moreover, it could also provide new information on the molecular and genetic mechanisms that affect the onset and progression of aggressive periodontitis in these cases (stages III/IV, grade C periodontitis) [1].

Taken together, the studies analyzed underscore that periodontal disease in the context of genetic syndromes with immunological alterations cannot be explained solely by dental plaque accumulation. Instead, it should be understood as a multifactorial process in which the genetically determined immune response plays a decisive role. While these findings suggest potential implications for more individualized therapeutic strategies that extend beyond biofilm control, such translational interpretations remain preliminary. A feasible path toward clinical translation may include the validation of these molecular profiles as diagnostic or prognostic biomarkers, their use for patient stratification in precision trials, and the exploration of targeted host-modulatory therapies (e.g., IL-17 or IFN/JAK-STAT inhibitors). Further mechanistic and clinical research is required before personalized interventions—such as modulation of specific inflammatory pathways (e.g., IL-17 in LAD-I or interferon signaling in DS)—can be reliably implemented in clinical practice [38].

Beyond the associations described, mechanistic evidence from experimental and translational studies helps to clarify the potential causal direction of these findings. In Down syndrome, the chronic overactivation of the IFN/JAK-STAT pathway has been shown to precede the development of pro-inflammatory cascades and to modulate myeloid cell function, suggesting a driving role in the exaggerated immune response that contributes to tissue breakdown [39,40,41]. Conversely, in LAD-I, the marked upregulation of IL-17 and related cytokines appears to represent a compensatory mechanism secondary to defective neutrophil migration, which subsequently amplifies local inflammation [6,42]. Similarly, in Papillon–Lefèvre syndrome, cathepsin C deficiency leads to impaired activation of neutrophil serine proteases and reduced LL-37 production, representing a primary genetic defect that predisposes to ineffective microbial clearance and sustained inflammation [43,44,45]. Together, these mechanistic insights support the view that, while certain molecular pathways may act as initiators of the pathological process, others may arise as consequences of immune dysregulation and chronic bacterial challenge, both of which contribute to the distinct periodontal phenotypes observed across these syndromes.

Finally, although the evidence presented is compelling, it must be interpreted with caution due to methodological limitations, including small sample sizes and heterogeneity in clinical and molecular parameters among the studies. Future research with larger cohorts and longitudinal designs will be necessary to better delineate the role of genetic and immunological mediators in periodontal susceptibility. The inclusion of only a small number of studies (n = 6) is another prominent limitation. In this way, this may reflect the complexity and rarity of these diseases that restrict the ability to perform quantitative meta-analyses and limit the generalizability of the findings. In addition, an important part of the existing literature consists of case reports or clinical series that, although they provide valuable information, do not meet the methodological criteria required for inclusion in systematic reviews.

## 5. Conclusions

This systematic review shows that patients with periodontitis associated with genetic or immune-mediated disorders present inflammatory gene expression profiles distinct from those with periodontitis without systemic conditions. Alterations in specific inflammatory genes and biomarkers were identified that contribute to increased disease susceptibility and progression. These findings underscore the importance of understanding particular immunoinflammatory mechanisms to guide personalized therapeutic strategies. However, further studies with large samples and homogeneous designs are required to obtain consistent and clinically applicable results.

## Figures and Tables

**Table 1:** Questions on patients, intervention, comparison, outcome, and studies (PICOS).

	Included	Excluded
Patients	Patients with periodontitis associated with genetic immunological disorders and those with periodontitis but no systemic conditions.	Patients with periodontitis unrelated to genetic or immunological disorders
Interventions	Investigate inflammatory gene expression in patients with periodontitis associated with genetic disorders or immunological diseases, as classified by the American Academy of Periodontology (AAP). Molecular markers, cytokines, or other inflammatory mediators measured in gingival tissue, gingival crevicular fluid, saliva, or blood samples	Studies that do not analyze inflammatory gene expression in periodontitis. Research focused on non-genetic or non-immunological systemic conditions (e.g., diabetes, cardiovascular diseases) without a clear link to the AAP classification.
Comparison	Patients with chronic periodontitis but without any known systemic diseases or genetic disorders	Participants with known genetic or immunological diseases, smokers, or those with other factors influencing inflammation or periodontal health
Outcome	Analyze differences in the expression of inflammatory genes between affected patients and those with periodontitis but without systemic disorders, providing insights into the molecular mechanisms underlying disease progression	
Studies	Cross-Sectional Studies Case–Control Studies Cohort Studies Clinical Trials Randomized Controlled Trials (RCTs)	Case reports Expert opinions Narrative reviews

## Data Availability

The developed protocol was previously registered and allocated the identification number CRD420251014916 in PROSPERO, the International Prospective Register of Systematic Reviews database, hosted by the National Institute for Health Research (NIHR), Centre for Reviews and Dissemination, University of York, York (UK) (www.crd.york.ac.uk/PROSPERO, accessed on 19 March 2025).

## Supplementary Materials

The following supporting information can be downloaded at: https://www.mdpi.com/article/10.3390/biomedicines13122851/s1. Table S1: PRISMA 2020 Checklist. Reference [46] is cited in Supplementary materials.
