# Supplementary material for: Differences in Inflammatory Genetic Profiles in Periodontitis Associated with Genetic and Immunological Disorders: A Systematic Review
Source: Biomedicines. 2025 Nov 21;13(12):2851. doi: 10.3390/biomedicines13122851 (PMC12730983; doi:10.3390/biomedicines13122851)
Supplement: Supplementary file 1 [file biomedicines-13-02851-s001.zip › biomedicines-3965833-supplementary.pdf]

PRISMA 2020 Checklist

Section and Topic	Item #	Checklist item	Location where item is reported
**TITLE**			
Title	1	Identify the report as a systematic review.	Title: 'Differences in inflammatory genetic profiles in periodontitis associated with genetic and immunological disorders: a systematic review'.
**ABSTRACT**			
Abstract	2	See the PRISMA 2020 for Abstracts checklist.	Abstract: Structured summary following PRISMA 2020, including background, methods, results, and conclusions.
**INTRODUCTION**			
Rationale	3	Describe the rationale for the review in the context of existing knowledge.	Introduction, paragraphs 1–3: Describes rationale within existing knowledge on genetic and immune-related periodontitis.
Objectives	4	Provide an explicit statement of the objective(s) or question(s) the review addresses.	Introduction, final paragraph: Explicit research questions provided (section 2.1 Focused review questions).
**METHODS**			
Eligibility criteria	5	Specify the inclusion and exclusion criteria for the review and how studies were grouped for the syntheses.	Methods, section 2.3:

Section and Topic	Item #	Checklist item	Location where item is reported
			Inclusion/exclusion criteria detailed and grouped using PICOS framework (Table 1).
Information sources	6	Specify all databases, registers, websites, organisations, reference lists and other sources searched or consulted to identify studies. Specify the date when each source was last searched or consulted.	Methods, section 2.4: Databases searched (PubMed, Scopus, Web of Science, Embase) and search date range (2010–June 2025).
Search strategy	7	Present the full search strategies for all databases, registers and websites, including any filters and limits used.	Methods, section 2.4: Search strategy with MeSH terms and free-text combinations described.
Selection process	8	Specify the methods used to decide whether a study met the inclusion criteria of the review, including how many reviewers screened each record and each report retrieved, whether they worked independently, and if applicable, details of automation tools used in the process.	Methods, section 2.5: Study selection performed independently by two reviewers with kappa agreement values.
Data collection process	9	Specify the methods used to collect data from reports, including how many reviewers collected data from each report, whether they worked independently, any processes for obtaining or confirming data from study investigators, and if applicable, details of automation tools used in the process.	Methods, section 2.5: Data extraction by two independent reviewers with resolution by discussion or third reviewer.
Data items	10a	List and define all outcomes for which data were sought. Specify whether all results that were compatible with each outcome domain in each study were sought (e.g. for all measures, time points, analyses), and if not, the methods used to decide which results to collect.	Methods, section 2.3 and 3.2:

Section and Topic	Item #	Checklist item	Location where item is reported
			Outcomes defined as inflammatory gene expression profiles and cytokine levels.
	10b	List and define all other variables for which data were sought (e.g. participant and intervention characteristics, funding sources). Describe any assumptions made about any missing or unclear information.	Methods, section 2.3: Other variables include patient characteristics, study design, molecular markers, and sample type.
Study risk of bias assessment	11	Specify the methods used to assess risk of bias in the included studies, including details of the tool(s) used, how many reviewers assessed each study and whether they worked independently, and if applicable, details of automation tools used in the process.	Methods, section 2.6: Risk of bias assessed using Cochrane ROBINS-E tool by two reviewers.
Effect measures	12	Specify for each outcome the effect measure(s) (e.g. risk ratio, mean difference) used in the synthesis or presentation of results.	Methods, section 2.7: Effect measures described narratively, not quantitatively synthesized.
Synthesis methods	13a	Describe the processes used to decide which studies were eligible for each synthesis (e.g. tabulating the study intervention characteristics and comparing against the planned groups for each synthesis (item #5)).	Methods, section 2.7: Synthesis methods described; studies classified by genetic disorder (DS, LAD-I, PLS).
	13b	Describe any methods required to prepare the data for presentation or synthesis, such as handling of missing summary statistics, or data conversions.	Methods, section 2.7: Handling of missing data and narrative synthesis approach described.

Section and Topic	Item #	Checklist item	Location where item is reported
	13c	Describe any methods used to tabulate or visually display results of individual studies and syntheses.	Results, Table 2: Tabulated study characteristics and outcomes.
	13d	Describe any methods used to synthesize results and provide a rationale for the choice(s). If meta-analysis was performed, describe the model(s), method(s) to identify the presence and extent of statistical heterogeneity, and software package(s) used.	Methods, section 2.7: Narrative synthesis justified due to heterogeneity; no meta-analysis performed.
	13e	Describe any methods used to explore possible causes of heterogeneity among study results (e.g. subgroup analysis, meta-regression).	Methods, section 2.7: Subgroup analyses mentioned (age, gender, genetic disease).
	13f	Describe any sensitivity analyses conducted to assess robustness of the synthesized results.	Methods, section 2.7: Sensitivity analysis conducted for robustness assessment.
Reporting bias assessment	14	Describe any methods used to assess risk of bias due to missing results in a synthesis (arising from reporting biases).	Methods, section 2.6: Risk of bias due to missing results evaluated within ROBINS-E framework.
Certainty assessment	15	Describe any methods used to assess certainty (or confidence) in the body of evidence for an outcome.	Methods, section 2.6 and 3.3: Certainty in evidence qualitatively discussed.
**RESULTS**			
Study selection	16a	Describe the results of the search and selection process, from the number of records identified in the search to the number of studies included in the review, ideally using a flow diagram.	Results, section 3.1

Section and Topic	Item #	Checklist item	Location where item is reported
			and Figure 1: PRISMA 2020 flow diagram describing selection process.
	16b	Cite studies that might appear to meet the inclusion criteria, but which were excluded, and explain why they were excluded.	Results, section 3.1: Reasons for exclusion defined during second-stage screening.
Study characteristics	17	Cite each included study and present its characteristics.	Results, Table 2: Characteristics of included studies.
Risk of bias in studies	18	Present assessments of risk of bias for each included study.	Results, Figure 2: Risk of bias for each included study (ROBINS-E).
Results of individual studies	19	For all outcomes, present, for each study: (a) summary statistics for each group (where appropriate) and (b) an effect estimate and its precision (e.g. confidence/credible interval), ideally using structured tables or plots.	Results, section 3.2: Individual study results summarized (gene expression and cytokine levels).
Results of syntheses	20a	For each synthesis, briefly summarise the characteristics and risk of bias among contributing studies.	Results, section 3.3: Summary of characteristics and bias among contributing studies.
	20b	Present results of all statistical syntheses conducted. If meta-analysis was done, present for each the summary estimate and its precision (e.g. confidence/credible interval) and measures of statistical heterogeneity. If comparing groups, describe the direction of the effect.	Results, section 3.2: No meta-analysis performed; narrative results presented for all studies.
	20c	Present results of all investigations of possible causes of heterogeneity among study results.	Results, section 3.2: Exploration of heterogeneity by genetic disorder

PRISMA

PRISMA 2020 Checklist

Section and Topic	Item #	Checklist item	Location where item is reported
			type.
	20d	Present results of all sensitivity analyses conducted to assess the robustness of the synthesized results.	Methods, section 2.7: Sensitivity analysis for robustness described.
Reporting biases	21	Present assessments of risk of bias due to missing results (arising from reporting biases) for each synthesis assessed.	Results, section 3.3: Reporting bias assessed qualitatively (ROBINS-E).
Certainty of evidence	22	Present assessments of certainty (or confidence) in the body of evidence for each outcome assessed.	Results, section 3.3 and Discussion: Certainty of evidence discussed narratively.
**DISCUSSION**			
Discussion	23a	Provide a general interpretation of the results in the context of other evidence.	Discussion, section 4: Results interpreted in context of other evidence.
	23b	Discuss any limitations of the evidence included in the review.	Discussion, final paragraphs: Limitations of evidence acknowledged (small samples, heterogeneity).
	23c	Discuss any limitations of the review processes used.	Discussion, section 4: Limitations of review processes noted.
	23d	Discuss implications of the results for practice, policy, and future research.	Discussion, section 4: Implications for

PRISMA 2020 Checklist

Section and Topic	Item #	Checklist item	Location where item is reported
			personalized periodontics and future research discussed.
**OTHER INFORMATION**			
Registration and protocol	24a	Provide registration information for the review, including register name and registration number, or state that the review was not registered.	Methods, section 2.2: PROSPERO registration ID CRD420251014916.
	24b	Indicate where the review protocol can be accessed, or state that a protocol was not prepared.	Methods, section 2.2: Protocol registered and accessible in PROSPERO.
	24c	Describe and explain any amendments to information provided at registration or in the protocol.	No amendments to protocol reported.
Support	25	Describe sources of financial or non-financial support for the review, and the role of the funders or sponsors in the review.	Funding: Declared as no external funding (section Funding).
Competing interests	26	Declare any competing interests of review authors.	Conflicts of Interest: Authors declared no conflict (section Conflicts of Interest).
Availability of data, code and other materials	27	Report which of the following are publicly available and where they can be found: template data collection forms; data extracted from included studies; data used for all analyses; analytic code; any other materials used in the review.	Data Availability Statement: Protocol and data registration in PROSPERO; supplementary materials referenced.

From: Page MJ, McKenzie JE, Bossuyt PM, Boutron I, Hoffmann TC, Mulrow CD, et al. The PRISMA 2020 statement: an updated guideline for reporting systematic reviews. BMJ 2021;372:n71. doi: 10.1136/bmj.n71. This work is licensed under CC BY 4.0. To view a copy of this license, visit https://creativecommons.org/licenses/by/4.0/
